# AGO Austria recommendation on screening and diagnosis of Lynch syndrome (LS)

**DOI:** 10.1007/s00404-017-4392-y

**Published:** 2017-05-16

**Authors:** Alain G. Zeimet, Harald Mori, Edgar Petru, Stephan Polterauer, Alexander Reinthaller, Christian Schauer, Tonja Scholl-Firon, Christian Singer, Katharina Wimmer, Johannes Zschocke, Christian Marth

**Affiliations:** 1 Department of Obstetrics and Gynecology, Innsbruck Medical University, Anichstrasse 35, 6020 Innsbruck, Austria; 2 MFA-Wiener Medizinische Viktor Frankl Vereinigung, Erdbrustgasse 46, 1010 Vienna, Austria; 3 Department of Obstetrics and Gynecology, Medical University of Graz, Auenbruggerplatz 14, 8036 Graz, Austria; 4 Gynecologic Cancer Unit, Department for Gynecology and Gynecologic Oncology, Comprehensive Cancer Centre, Medical University Vienna, Währinger Gürtel 18-20, 1090 Vienna, Austria; 5 Department of Gynecology, Hospital Barmherzige Brüder Graz, Marschallgasse 12, 8020 Graz, Austria; 6 Department of Gynecology and Obstetrics, Wilhelminenspital, Montleart Straße 37, 1160 Vienna, Austria; 7 Division of Human Genetics, Innsbruck Medical University, Peter Mayr-Strasse 1, 6020 Innsbruck, Austria

**Keywords:** Austrian-AGO, Lynch syndrome, Endometrial cancer, Ovarian cancer, MSI, Mismatch repair

## Abstract

**Purpose:**

This manuscript reports the consensus recommendations on screening and diagnosis of Lynch syndrome (LS) in patients with endometrial or ovarian cancer as well as on possible preventive measures in effectively LS-diagnosed women. The recommendations are issued by the Austrian Arbeitsgemeinschaft für Gynäkologische Onkologie (AGO) of the Österreichischen Gesellschaft für Gynäkologie und Geburtshilfe (OEGGG) after consultation of the most recent and relevant literature and following deliberation by the Genetic Task-Force convoked May, 2015 by the AGO Council.

**Results and conclusion:**

The Austrian AGO recommends immunohistochemical tissue screening for type-I and type-II endometrial cancers in all patients below the age of 70 years, and for all endometrioid and clear-cell ovarian cancers independently of the patient’s age. If needed immunohistochemistry should be complemented by tissue MLH1 promotor hypermethylation testing and/or microsatellite instability (MSI) analysis. The diagnosis LS requires confirmation through identification of a germline mutation by a molecular genetic examination in the mismatch repair genes using the patient’s blood. This should be performed without preceding tissue screening when in LS-associated cancer patients the family history fulfills the Amsterdam II or the revised Bethesda criteria. In LS-diagnosed women, the age for prophylactic surgery should be set flexibly based on an informed consent. Regarding the monitoring of these women, chemo-preventive measures as well as screening procedures either to avoid or to early detect LS-related tumors are discussed with a special light on their specific limitations.

## Introduction

The autosomal dominant inherited tumor disposition syndrome first described by Henry Lynch in 1966 is caused by heterozygous (only one allele is affected) inactivating germline mutation in one of the mismatch repair (MMR) genes *MLH1, MSH2, MSH6* or *PMS2.* LS may also be due to a germline deletion of the 3′ end of the *EPCAM* gene, which causes epigenetic inactivation of the neighboring *MSH2* gene. Somatic loss of the second allele in neoplastic cells either, through monoallelic promotor hypermethylation, gene sequence alteration, a large deletion or another genetic alteration leading to loss of the heterozygosity (LOH) causes expression loss of the respective MMR protein and, consequently, DNA MMR deficiency. Intratumoral loss of one of these repair proteins is detected with immunohistochemistry (IHC) and the molecular correlate of MMR deficiency is microsatellite instability (MSI). Microsatellites are DNA sequences spread throughout the genome; their repetitive sequence structure makes them susceptible for replication errors that can only be repaired with a functioning MMR system. Changes in microsatellites consequently serve as markers for a non-functioning MMR system in LS.

However, loss of MMR function is also observed in some non-LS-related, sporadic endometrial cancers and is most frequently caused by loss of the MLH1 protein due to biallelic somatic hypermethylation of the *MLH1* promotor.

Individuals with LS have, depending on which MMR gene is mutated, a lifetime risk of 20–75% of developing a colorectal carcinoma. That is why the syndrome previously was called hereditary nonpolyposis colorectal cancer (HNPCC). Women have an almost equally high lifetime risk for developing endometrial cancer [1]. The endometrial carcinoma is frequently observed in women as an initial malignancy (so-called sentinel malignancy) that precedes a colorectal carcinoma diagnosis [2]. Women with LS also have a life-long risk of up to 12% for developing ovarian cancer, which may occur either alone or synchronously with another LS-associated malignancy. LS-related ovarian carcinomas are endometrioid or clear-cell carcinomas or histological mixed forms with predominance of the two mentioned histological components. Purely serous or mucinous histological subtypes are not to be expected. In addition, individuals with LS also have a higher risk for other malignancies such as those of the stomach, small intestine, hepatobiliary epithelium, urothelium and brain. Thus, it is essential to obtain a detailed patient and family history for all cancer patients [3].

Of all diagnosed endometrial carcinomas, between 1.8 and 3% are associated with LS, while this is the case for 2.8% of colorectal carcinomas. Median age at diagnosis of LS-associated endometrial carcinoma is between 47 and 55 years, depending on the studied cohort. However, a notably high percentage of >30% of cases in women are diagnosed after the age of 60. Especially MSH6-associated endometrial carcinomas occur comparatively later [1, 4, 5]. In addition, 8–20% of all endometrioid or clear-cell ovarian carcinomas exhibit microsatellite instability [6] and are LS associated. They generally occur at a younger age (median age 47 years) compared to non-LS-associated ovarian cancers [7].

## Diagnosis of LS

Suspicion for LS may derive from the patient’s and her family’s cancer history or from specific tumor characteristics. International criteria based on the personal or the family history have been established to select individuals who are at high risk for LS. The Amsterdam I (1991) and Amsterdam II (1999) criteria primarily refer to the frequent occurrence of LS-typical carcinomas in several closely related members of a family (at least three LS patients in at least two generations, age at diagnosis <50 years in at least one person) (Table 1) [8]. The Bethesda criteria revised in 2002 give recommendations on the specific analysis of the tumor tissue for particular constellations (age at diagnosis, histology, multiple tumors, etc.) (Table 2) [9]. However, historically the main focus of these recommendations was the prevention of colorectal carcinomas [1]. Retrospective evaluations have shown that precisely in the case of LS-associated endometrial carcinomas the mentioned criteria often do not permit proper risk assessment and women at increased risk of endometrial cancer do not consistently fulfill selection criteria [4].

**Table 1:** Amsterdam II criteria

There should be at least three relatives with a Lynch/HNPCC-associated cancer (cancer of the colorectum, endometrium, small bowel, ureter or renal pelvis) and…
One should be a first-degree relative to the other two At least two successive generations should be affected At least one should be diagnosed before age 50 Familial adenomatous polyposis should be excluded Tumors should be verified by pathological examination

Vasen et al. [8]

**Table 2:** Revised Bethesda guidelines

Tumors from individuals should be tested for MSI in the following situations
1. Colorectal cancer diagnosed in a patient who is less than 50 years of age 2. Presence of synchronous, metachronous colorectal or other HNPCC-associated tumors^a^, regardless of age 3. Colorectal cancer with the MSI-high histology^b^ diagnosed in a patient who is younger than 60 years of age 4. Colorectal cancer diagnosed in one or more first-degree relatives with an HNPCC-related tumor^a^, with one of the cancers being diagnosed before age 50 years 5. Colorectal cancer diagnosed in two or more first- or second-degree relatives with HNPCC-related tumors, regardless of age

Umar et al. [9]

^a^HNPCC-related tumors include colorectal, endometrial, gastric, ovarian, pancreatic, ureter/renal pelvis, biliary tract and brain (usually glioblastoma as seen in Turcot syndrome) tumors, sebaceous gland adenomas and keratoacanthomas in Muir–Torre syndrome, and carcinoma of the small bowel

^b^Presence of tumor infiltrating lymphocytes, Crohn’s-like lymphocytic reaction, mucinous/signet-ring differentiation or medullary growth pattern

There are two tumor tissue-based specific screening procedures for raising strong suspicion of LS from:

- Immunohistochemical detection of the absence of one of the four relevant MMR proteins MLH1, MSH2, MSH6 or PMS2 in tumor tissue.
- Identification of MSI by analyzing five defined microsatellites with RT-PCR from tumor DNA; two or more positive markers indicate a very strong suspicion of LS.

**Fig. 1:**
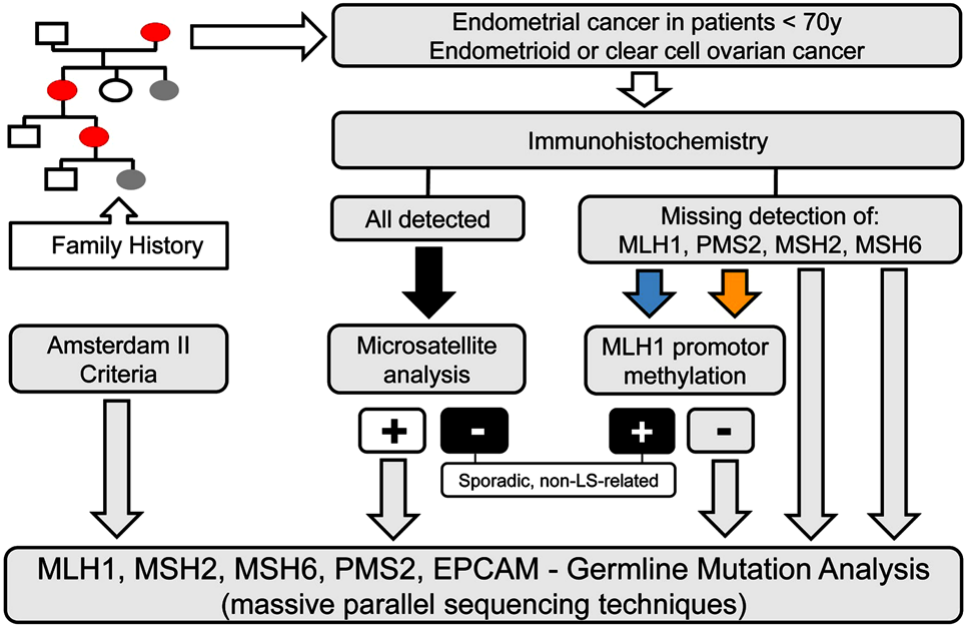
Flow chart for Lynch syndrome work-up

In case of immunohistochemical absence of either MLH1 or PMS2 [10], or demonstrated microsatellite instability, tumor tissue should be tested for biallelic hypermethylation of the *MLH1* promotor, which is indicative for sporadic non-LS-associated cancers. In 25% of all diagnosed endometrial cancers, MLH1 is missing on immunohistochemistry, and in 75% of these cases this is due to biallelic hypermethylation of the MLH1 promotor. In the less frequent cases of isolated loss of PMS2 immunostaining (2.5%), also MLH1 promotor hypermethylation is causative in 50% of the cases [10]. Therefore, methylation status of *MLH1* promotor should also be tested in these cancers (Fig. 1).

However, in patients with a manifest LS-associated carcinoma and a positive family anamnesis according to the Amsterdam II or the revised Bethesda 2002 criteria the mentioned screening procedure should be skipped and a germline analysis should be offered immediately.

The diagnosis “Lynch syndrome” requires confirmation through identification of a germline mutation by means of a molecular genetic examination of the MMR gene(s) using the patient’s blood. *Gene*-*targeted molecular germline testing* is based usually on the immunohistochemical results of missing proteins. For this selective testing, it should be emphasized that it is important to include deletion of the 3′ end of the *EPCAM* gene if there is a loss of MSH2 protein expression. Furthermore, *MLH1* germline mutation was identified in 23% of cancers with isolated immunohistochemical PMS2 loss. Therefore, analysis of the *MLH1* mutational status should be advocated in addition to that of *PMS2* in these cases [11]. However, massive parallel sequencing techniques presently allow analysis of all relevant MMR genes simultaneously with the highest cost effectiveness (Fig. 1).

In Austria, all molecular genetic testing for germline mutations requires written informed consent following detailed genetic counseling according to § 69, of the Austrian Gentechnik-Gesetz.

Currently, evidence of LS has no direct therapeutic consequences but serves to predict the risk for synchronous and metachronous LS-associated malignancies in the patient herself or in her direct relatives. In the near future, a therapeutic consequence may arise from the MMR status in ovarian and endometrial cancers. Better response to immunotherapy with checkpoint inhibitors (e.g., PD1 antibodies) has been demonstrated in LS-associated colon carcinomas as compared with microsatellite-stable cancers and this may also prove true for other LS-associated tumors [12].

## AGO Austria recommendations

In agreement with the NCCN guidelines 2014 [13], the AGO Austria recommends for all women with endometrial carcinoma (types I and II) below age of 70 years that tumor tissue is tested for LS. The recommended method is immunohistochemical analysis for the MMR proteins MLH1, MSH2, MSH6 and PMS2 in a first step. If immunohistochemistry is unremarkable (nuclear expression of all MMR proteins), but the patient’s personal or family history is suggestive for LS (i.e., one first-degree relative with an LS-associated malignancy or another LS-associated malignancy in the patient’s own history), LS screening should be expanded to microsatellite instability analysis as some mutations may give rise to stable non-functioning proteins that are missed by immunohistochemistry [1]. In addition, AGO Austria recommends that these analyses should also be conducted irrespective of age at diagnosis for endometrioid and clear-cell ovarian cancers.

If one or more of the relevant MMR proteins cannot be identified, or microsatellite instability is demonstrated after exclusion of MLH1 promotor hypermethylation, molecular genetic testing for germline mutation should be offered after previous genetic counseling according to § 69, of the Austrian Gentechnik-Gesetz.

Even if all screening results are negative but the family history is nevertheless strongly suggestive for LS (i.e., Amsterdam II or revised Bethesda criteria are met) molecular genetic testing of all known LS-relevant genes should be considered [4, 13].

With regard to cancer prophylaxis and early detection in women with proven LS, AGO Austria in agreement with other medical societies recommends:

1. Prophylactic total hysterectomy with bilateral salpingo-oophorectomy from age 35 years or after completed family planning [14] should be discussed. The patient is to be informed that bilateral salpingectomy alone is not sufficient with regard to the histological subtypes of ovarian cancers, which could be expected. The age limit for prophylactic surgery should be set flexibly on the basis of a personalized decision made with the patient under consideration of the earliest age at diagnosis in the family history and her own endocrinological preferences. For orientation in this purpose, the following key parameters from the observational study by Schmeler et al. can be used [13]. In the non-interventional, i.e., only observed subgroup of LS women, endometrial cancer occurred in 33% and the median age at diagnosis was 46 (range 30–60) years. However, 6% of these women (corresponding to 2% of all LS women of the non-interventional subgroup) were of age 35 or younger at diagnosis, and 18% (i.e., 6% of the non-interventional subgroup) were of age 40 or younger at diagnosis. In the non-interventional group, ovarian cancer was less frequently diagnosed (5.5%), and the median age at diagnosis was 42 (range 31–48) years. Of these patients, 17% (i.e., 1% of the non-interventional subgroup) were of age 35 or younger and 37% (i.e., 2.3% of the entire non-interventional subgroup) were 40 years or younger [15].

Of special note is that according to the current state of the art, hormone replacement therapy can be offered to these women following salpingo-oophorectomy.

2. Women who wish to avoid the risks of surgery and premature menopause and who understand the risk of ovarian- and endometrial cancer and the lack of efficient screening for early detection of both cancers might nevertheless choose observation. As an alternative, the patient should be offered the possibility of an annual endometrium biopsy (pipelle eventually complemented by an office-hysteroscopy) together with transvaginal ultrasound examination from age 30/35 years [13, 14, 16].
3. To prevent colorectal cancer: regular colonoscopy at intervals of 1–2 years from age 20/25 years, or 10 years before the earliest occurrence of a colorectal carcinoma in the family anamnesis, should be performed.

Moreover, chemoprevention by means of NSRA or acetylsalicylic acid can prevent the occurrence of colorectal carcinomas [17].

4. Regular medical examinations for early detection for other LS-relevant tumors.
5. Inform the patient about the symptoms of the mentioned malignancies and the need to explore such symptoms earliest if they occur.
6. Chemoprevention using oral contraceptives can be considered for young women, even though there are no prospective studies that demonstrate their efficacy for women with LS in particular.
